# Construction of CoP_2_-Mo_4_P_3_/NF Heterogeneous Interfacial Electrocatalyst for Boosting Water Splitting

**DOI:** 10.3390/nano13010074

**Published:** 2022-12-23

**Authors:** Yafeng Chen, Ge Meng, Ziwei Chang, Ningning Dai, Chang Chen, Xinmei Hou, Xiangzhi Cui

**Affiliations:** 1 The State Key Laboratory of High Performance Ceramics and Superfine Microstructures, Shanghai Institute of Ceramics, Chinese Academy of Sciences, Shanghai 200050, China; 2 Beijing Advanced Innovation Center for Materials Genome Engineering, Collaborative Innovation Center of Steel Technology, University of Science and Technology Beijing, Beijing 100083, China; 3 Shanghai Motor Vehicle Inspection Certification & Tech Innovation Center Co., Ltd., Shanghai 201805, China; 4 School of Chemistry and Materials Science, Hangzhou Institute for Advanced Study, University of Chinese Academy of Sciences, Hangzhou 310021, China

**Keywords:** heterogeneous interface, transition metal phosphide, bifunctional electrocatalyst, overall water splitting

## Abstract

Developing highly efficient, cost effective and durable bifunctional electrocatalyst remains a key challenge for overall water splitting. Herein, a bifunctional catalyst CoP_2_-Mo_4_P_3_/NF with rich heterointerfaces was successfully prepared by a two-step hydrothermal-phosphorylation method. The synergistic interaction between CoP_2_ and Mo_4_P_3_ heterogeneous interfaces can optimize the electronic structure of active sites, leading to the weak adsorption of H on the Mo sites and the increased redox activity of the Co site, resultantly improving the HER/OER bifunctional catalytic activity. The synthesized CoP_2_-Mo_4_P_3_/NF catalyst exhibits excellent electrocatalytic activity in 1.0 M KOH with low overpotentials of 77.6 and 300.3 at 100 mA cm^−2^ for HER and OER, respectively. Additionally, the assembled CoP_2_-Mo_4_P_3_/NF||CoP_2_-Mo_4_P_3_/NF electrolyzer delivers a current density of 100 mA cm^−2^ at a cell voltage of 1.59 V and remains stable for at least 370 h at 110 mA cm^−2^, indicating the potential application prospective in water splitting.

## 1. Introduction

As a clean and sustainable energy, hydrogen (H_2_) has shown great promise to replace traditional fossil fuels [1,2,3]. Electrocatalytic overall water splitting (OWS), including hydrogen evolution reaction (HER) at cathode and oxygen evolution reaction (OER) at anode, has attracted huge attention as a key technology for high purity hydrogen production without additional by-products [4,5]. However, the practical application of large-scale hydrogen production through water splitting is limited by the thermodynamically uphill processes of HER and OER [6,7]. Therefore, electrocatalysts with high activity and good stability are desired to effectively accelerate the reaction kinetics and reduce the reaction overpotential, consequently reducing the electric consumption. To date, the state-of-the-art catalysts for HER and OER are mainly noble metal materials (Pt for HER and Ru/Ir oxides for OER, respectively), which faces the problems of high cost, poor stability, low energy conversion efficiency and low bifunctionality [8,9]. Therefore, the development of efficient, inexpensive and stable nonprecious metal-based electrocatalysts remains a critical issue to be solved in water splitting.

Recently, transition metal phosphides (TMPs) have received numerous attentions as electrocatalysts for water splitting in alkaline electrolytes, due to their high conductivity, high catalytic activity and expected durability among wide pH value [10,11,12]. The negatively charged P in TMPs can not only provide high activity for the formation and desorption of H_2_, but also enhance the density of states around the Fermi level, which provides the prerequisite for it to be a bifunctional catalyst for HER and OER [13,14,15]. Particularly, Molybdenum-based phosphides (Mo_x_P_y_) exhibit prominent advantages as efficient electrocatalysts because of their metallic characteristics, superior stability and the similar d-band electronic structure to the noble metal platinum. However, the relevant catalytic activity of Mo_x_P_y_ is still far from that of noble metal-based catalysts, and its bifunctional catalytic activity still needs further improvement. Currently, great efforts have been devoted to enhance the electrocatalytic activity of Mo_x_P_y_ catalysts by regulating the number of active sites, electron structure, conductivity and the electron transfer capability [16,17,18]. For instance, Jiang et al. [13] fabricated porous MoP nanoflake array grown on nickel foam (MoP/NF) as bifunctional electrocatalysts for water splitting, which exhibited high electrochemical surface area and number of active sites on the surface, resulting in excellent activity for overall water splitting with a cell voltage of 1.62 V at a current density of 10 mA cm^−2^. Xiao et al. [19] demonstrated a novel hybrid nanostructure composed of carbon encapsulating ultra-low Co/Ni-doped MoP nanoparticles, which could be adopted as highly active and stable HER catalysts in pH-universal electrolytes. The experimental and theoretical studies reveal that the doping of Co/Ni atoms in MoP results in effective charge transfer from Ni/Co to MoP, which facilitates the electron reconfiguration, enabling obvious changes in the hydrogen adsorption energy and a major improvement of HER performance. Despite persistent efforts, there is still a certain gap between the catalytic performance of Mo_x_P_y_ and noble metal-based catalysts. More importantly, the bifunctional catalytic activity of Mo_x_P_y_ still does not meet the requirements for industrial applications.

The synergistic effects between multi-component interfaces of heterogeneous structures can not only promote the charge transfer and optimize the electronic structure, but also give full play to the physicochemical properties and synergistic catalytic ability of the composite catalysts, thus effectively improving the catalytic activity of the heterogeneous structure [20]. With these in mind, herein, we designed a novel CoP_2_-Mo_4_P_3_/NF catalyst with rich heterointerfaces by a two-step hydrothermal-phosphorylation method, which proved to be an efficient bifunctional catalyst for the overall water splitting in alkaline electrolyte. The experimental results show that the synergistic effect between CoP_2_ and Mo_4_P_3_ heterogeneous interfaces can not only effectively improve the electrochemical active area and reduce the surface charge transfer impedance, but also optimize the electronic structure of the active sites, thus improving the bifunctional intrinsic catalytic activity of the composite. The obtained CoP_2_-Mo_4_P_3_/NF catalyst reveals excellent HER/OER bifunctional catalytic activity under alkaline conditions, with an overpotential of 77.1 mV (HER) and 300.3 mV (OER) at a current density of 100 mA cm^−2^, respectively. Additionally, the obtained CoP_2_-Mo_4_P_3_/NF catalyst need only 1.59 V and 1.80 V to reach the current densities of 100 and 500 mA cm^−2^ in the two-electrode electrocatalytic water splitting system, and the corresponding catalytic activity does not deteriorate obviously after 370 h continuous reaction at 110 mA cm^−2^, indicating the potential industrial application prospective in water splitting.

## 2. Materials and Methods

### 2.1. Materials

Hexaammonium heptamolybdate tetrahydrate ((NH_4_)_6_Mo_7_O_24_∙4H_2_O, 99.0%), Cobaltous nitrate hexahydrate (Co(NO_3_)_2_∙6H_2_O, 98.5%) and Hydrochloric acid (HCl, AR, ~40%) were purchased from Sinopharm Group Chemical Reagent Co., Ltd., China. Sodium dodecyl sulfate (SDS, C_12_H_25_SO_4_Na) and Sodium hypophosphite (NaH_2_PO_2_, 99.0 wt.%) were purchased from Aladdin, Shanghai, China. Commercial Pt/C (Pt 20 wt.%) was purchased from Shanghai HEPHAS Energy Equipment Co., Ltd., Shanghai, China. Nickel foam (NF, thickness: 1.5 mm) was purchased from Cyber Electric Co., Ltd., China.

### 2.2. Sample Synthesis

#### 2.2.1. Synthesis of Co-MoO_x_/NF and MoO_x_/NF

Firstly, prior to the synthesis, the NF (2 × 4 cm^2^) was pre-treated with HCl (3M), ethanol and DI water by ultrasonication for 15 min, successively. Then, 0.7 mM (NH_4_)_6_Mo_7_O_24_∙4H_2_O and 0.49 mM Co(NO_3_)_2_∙6H_2_O were dissolved in 30 mL of DI water. After stirring for 10 min, 1 g SDS was added into the solution and stirred strongly until the SDS dissolved completely, and a piece of cleaned NF was placed into the solution. Afterward, the solution was transferred into a 40 mL Teflon-lined autoclave and hydrothermally heated at 150 °C for 8 h. After cooling to room temperature, the product was rinsed by ethanol and DI water for several times and the dried at 60 °C for 12 h under vacuum to obtain the Co-MoO_x_/NF precursor. The MoO_x_/NF was prepared with the same procedure without the addition of Co(NO_3_)_2_∙6H_2_O.

#### 2.2.2. Synthesis of CoP_2_-Mo_4_P_3_/NF and Mo_4_P_3_/NF

The CoP_2_-Mo_4_P_3_/NF was synthesized by one-step phosphorization of the Co-MoO_x_/NF precursor. NaH_2_PO_2_ (1 g) was used as P source and placed at the upstream of a tube furnace, and the Co-MoO_x_/NF was placed at the middle of the tube furnace. Then, they were heated up to 500 °C and kept for 2 h in Ar gas flow with a rate of 5 °C min^−1^. After cooling to room temperature, the CoP_2_-Mo_4_P_3_/NF catalyst was obtained. The synthesis procedure of Mo_4_P_3_/NF is the same as that of CoP_2_-Mo_4_P_3_/NF.

#### 2.2.3. Materials Characterization

X-ray diffraction (XRD) tests were performed on a Rigaku (Japan) D/Max-2550 V X-ray diffractometer at a scan rate of 5° min^−1^ (λ = 0.154 nm, 40 kV, 40 mA). The morphologies of the synthesized catalysts were obtained by scanning electron microscope (SEM, ThermoFisher Scientific, Quattro S, USA). Transmission electron microscopy (TEM), high-resolution transmission electron microscopy (HRTEM), energy dispersive X-ray spectrometer (EDS) and selected area electron diffraction (SAED) were carried out using a FEI talos F200x G2 field emission transmission electron microscopy (200 kV). X-ray photoelectron spectroscopy (XPS) measurements were performed with a Thermo Scientifc K-Alpha+ using a monochromatic Al Kα source (15 kV, 15 mA) and the binding energies were referenced to C 1s peak at 284.8 eV.

#### 2.2.4. Electrochemical Measurements

All the electrochemical measurements of the synthesized samples were performed at room temperature on a CHI 760E (CH instruments, Inc., Shanghai) electrochemical workstation in 1.0 M KOH (pH = 14) electrolyte. The as-obtained catalysts grown on the NF, Ag/AgCl electrode saturated with KCl solution and graphite rod were used as the working, reference and counter electrodes, respectively. All the measured potentials against Ag/AgCl electrode were converted to potentials referenced of the reversible hydrogen electrode (RHE) according to the Nernst Equation (E_RHE_ = E_Ag/AgCl_ + 0.0592 × pH + 0.1989 V). HER and OER measurements were carried out in a standard three-electrode system, while the overall water splitting (OWS) was in a two-electrode system.

Before electrochemical measurements, the electrolyte was saturated with high-purity N_2_ for 30 min. In total, 40 cyclic voltammetry (CV) cycles with a sweep rate of 50 mV s^−1^ were performed to bubble away the surface contaminates and stabilize the catalysts. Then, the linear sweep voltammetry (LSV) curves were obtained at a scan rate of 2 mV s^−1^ with 100% iR compensations. The Tafel slopes were calculated by Tafel equation (η = a + b log(j)). The electrochemical impedance spectroscopy (EIS) measurements were carried out at −0.03 V (vs. RHE) for HER and 1.49 V (vs. RHE) for OER from 0.01 Hz to 100 KHz. The double layer capacitance (C_dl_) was calculated from CV curves at different scan rates of in a non-Faraday area. The electrochemically active surface areas (ECSAs) were calculated based on the equation: ECSA = C_dl_/C_s_, where Cs is the specific capacitance (C_s_ = 40 μF cm^−2^ was used in this work). Chronoamperometric measurements (CA) was performed to assess the stability and durability of the obtained catalysts.

## 3. Results and Discussion

### 3.1. Catalyst Synthesis and Characterization

The synthesis of Co-MoO_x_/NF and MoO_x_/NF precursors were achieved by a facile hydrothermal method, and the XRD pattens of the obtained samples are shown in Figure S1a. The peaks at 22.0, 23.5, 27.3, 27.7, 29.3, 30.2, 33.4, 40.7 and 55.1° can be, respectively, assigned to the (010), (−111), (310), (−311), (−312), (−213), (601), (215) and (1000) crystal planes of Mo_9_O_26_ (PDF#05-0441), and those at 31.9, 42.1 and 53.7° can be, respectively, assigned to the (−201), (012) and (022) crystal planes of MoO_2_ (PDF#05-0452), indicating that MoO_x_ is the main phase of the precursors. Additionally, in the XRD patten of Co-MoO_x_/NF, the new peaks at 14.1, 43.4, 49.2 and 56.9° belonging to the (110), (023), (−114) and (043) planes of CoMoO_4_ (PDF#73-1331) can be observed, suggesting the successful introduction of Co element. After phosphorization treatment by NaH_2_PO_2_, the XRD pattens of the catalysts are shown in Figure S1b, where the diffraction peaks corresponding to Mo_4_P_3_ and CoP_2_ can be clearly observed. The diffraction peaks at 14.2, 26.1, 28.7, 29.9, 36.8, 41.0, 44.5, 47.3, 51.8 and 53.6° can be assigned to the (200), (006), (400), (206), (312), (411), (414), (604), (417) and (612) crystal planes of Mo_4_P_3_ (PDF#89-2586), respectively, indicating that the MoO_x_ precursor was transformed into Mo_4_P_3_ after phosphorization treatment. Moreover, the peaks at 32.2 and 66.9° can be, respectively, assigned to the (020) and (−204) planes of CoP_2_ (PDF#77-0263), which is due to the transformation of CoMoO_4_ in the Co-MoO_x_/NF precursor, suggesting the formation of CoP_2_-Mo_4_P_3_/NF catalyst with mixed phase of Mo_4_P_3_ and CoP_2_.

**Figure 1:**
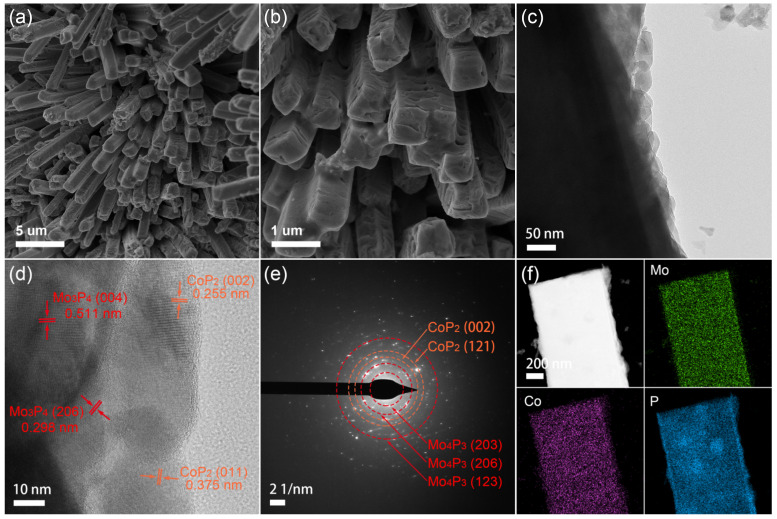
SEM (**a**,**b**), TEM (**c**), HRTEM (**d**), SAED (**e**) and the corresponding elemental mapping (**f**) images of CoP_2_-Mo_4_P_3_/NF catalyst.

SEM images show that both Co-MoO_x_/NF and MoO_x_/NF precursors exhibit a nanorod-like morphology with a smooth surface and an average rod diameter of 1 μm (Figure S2). After phosphorization treatment, the obtained CoP_2_-Mo_4_P_3_/NF and Mo_4_P_3_/NF catalysts still keep the rod-like morphology (Figure 1a and Figure S3a), while the surface of CoP_2_-Mo_4_P_3_/NF (Figure 1b) becomes rougher compared to that of Mo_4_P_3_/NF (Figure S3b), which is due to the generation of CoP_2_ on the CoP_2_-Mo_4_P_3_/NF surface. The TEM image in Figure 1c further manifests the rough surface of the CoP_2_-Mo_4_P_3_/NF catalyst. The HRTEM image in Figure 1d exhibits the lattice fringes with interplanar spacing of 0.511 and 0.298 nm, corresponding to the (004) and (206) planes of Mo_4_P_3_, respectively, while the interplanar spacing of 0.255 and 0.375 nm can be assigned to the (002) and (011) planes of CoP_2_, respectively. The diffraction rings corresponding to (203), (206) and (123) for Mo_4_P_3_ as well as (002) and (121) for CoP_2_ can also be observed in the SAED image (Figure 1e), confirming the existence of CoP_2_ and Mo_4_P_3_ phase in the CoP_2_-Mo_4_P_3_/NF catalyst. Moreover, the TEM-EDS line scanning image of CoP_2_-Mo_4_P_3_/NF shows that the P and Co elements are rich in the edge of the catalyst (Figure S4), while Mo elements are mainly distributed in the inner part, evidencing the presence of CoP_2_ particles on the surface of the Mo_4_P_3_ nanorods. Besides, the EDS elemental mapping in Figure 1f shows that the Mo, Co and P elements are homogeneously distributed in the CoP_2_-Mo_4_P_3_/NF catalyst, while Mo is slightly narrower than Co and P, further indicating the generation of CoP_2_ particles on the surface of the Mo_4_P_3_ nanorods. According to the previous study [21], the oxophilicity of Mo is much higher than that of Co, meaning that Co is more easily reduced during the phosphorylation process resultantly forming Co-P bonds. Therefore, with the increase of phosphorylation time and temperature, Co will combine with P firstly and form the phosphorus-rich CoP_2_ species. Then the MoO_x_ will be phosphatized to form the metal-rich Mo_4_P_3_ species resultantly forming heterogeneous interfaces with the surface CoP_2_. Additionally, the TEM, HRTEM, SAED and EDS measurements of the Mo_4_P_3_/NF catalyst are also performed for comparison. As illustrated in Figure S5, the surface of Mo_4_P_3_/NF is smoother than that of CoP_2_-Mo_4_P_3_/NF and only the Mo_4_P_3_ phase can be found.

**Figure 2:**
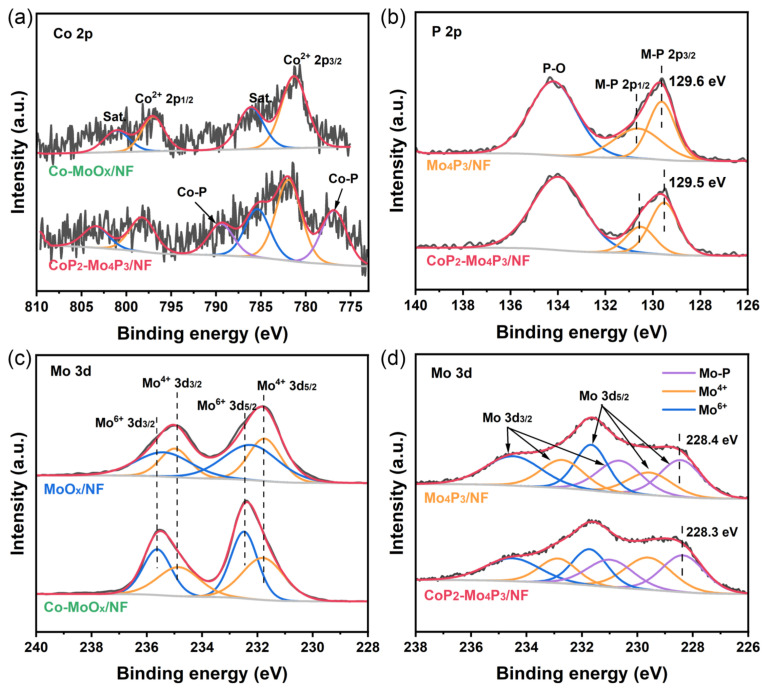
(**a**) Co 2p XPS spectra of Co-MoO_x_/NF and CoP_2_-Mo_4_P_3_/NF, (**b**) P 2p XPS spectra of Mo_4_P_3_/NF and CoP_2_-Mo_4_P_3_/NF, (**c**) Mo 3d XPS spectra of MoO_x_/NF and Co-MoO_x_/NF, (**d**) Mo 3d XPS spectra of Mo_4_P_3_/NF and CoP_2_-Mo_4_P_3_/NF.

To further investigate the surface compositions and the oxidation states of the elements in the resultant materials, the XPS measurements were performed. Figure 2a shows the high-resolution Co 2p spectra of Co-MoO_x_/NF and CoP_2_-Mo_4_P_3_/NF. The peaks at the binding energies of 781.4 and 797.1 eV can be respectively attributed to the Co 2p_3/2_ and 2p_1/2_ of Co^2+^, and the other two doublet peaks are satellite signals (785.7 and 800.5 eV). After phosphorization treatment, new peaks at 776.9 and 789.4 eV can be observed, corresponding to the Co^δ+^ of Co-P bond, indicating the generation of CoP_2_ phase [22]. The high-resolution P 2p spectra of Mo_4_P_3_/NF and CoP_2_-Mo_4_P_3_/NF are shown in Figure 2b, and the peak at 134.0 eV can be assigned to the P-O bond caused by the surface oxidation of metal phosphide [23]. Notably, the peaks at 129.5 and 130.5 eV correspond to the P 2p_3/2_ and 2p_1/2_ of P^δ−^ in the M-P bonds, demonstrating the successful formation of the metal phosphide phase [24]. In addition, the P^δ−^ peaks of CoP_2_-Mo_4_P_3_/NF are negatively shifted about 0.1 eV compared to those of Mo_4_P_3_/NF, being resulted from the charge transfer from Co to P atoms, further demonstrating the generation of CoP_2_.

Figure 2c reveals the high-resolution Mo 3d spectra of the MoO_x_/NF and Co-MoO_x_/NF precursors, where Mo is mainly present as Mo^4+^ and Mo^6+^, corresponding to MoO_2_ and Mo_9_O_26_ phases of the precursors, respectively [25]. The high-resolution Mo 3d spectra of Mo_4_P_3_/NF and CoP_2_-Mo_4_P_3_/NF are shown in Figure 2d. In addition to the peaks of Mo^4+^ and Mo^6+^, the peak at the binding energy of 228.3 eV corresponding to the Mo^δ+^ of Mo-P bond can also be detected, indicating the generation of Mo_4_P_3_ phase [26]. Moreover, the binding energy of Mo-P bond in CoP_2_-Mo_4_P_3_/NF (228.3 eV) is negatively shifted about 0.1 eV compared to that of Mo_4_P_3_/NF (228.4 eV), which may be caused by the electronic transfer from CoP_2_ to Mo_4_P_3_. Thus, the electron density of Mo atoms in the CoP_2_-Mo_4_P_3_/NF increase, leading to the weak adsorption of H on Mo sites and improving the HER catalytic activity. Meanwhile, the electron deficiency of CoP_2_ will result in a higher oxidation state of Co, leading to increased redox activity of the Co site and enhancing the catalytic activity of OER [27,28]. The above XPS analysis results manifest that the strong electronic coupling effects of the heterogeneous interfaces between CoP_2_ and Mo_4_P_3_ can optimize the electronic structure, modulate the adsorption of H on the Mo sites and increase the redox activity of the Co site, thus improving the bifunctional catalytic activity of the CoP_2_-Mo_4_P_3_/NF catalyst for both HER and OER.

### 3.2. Electrocatalytic HER Performance

**Figure 3:**
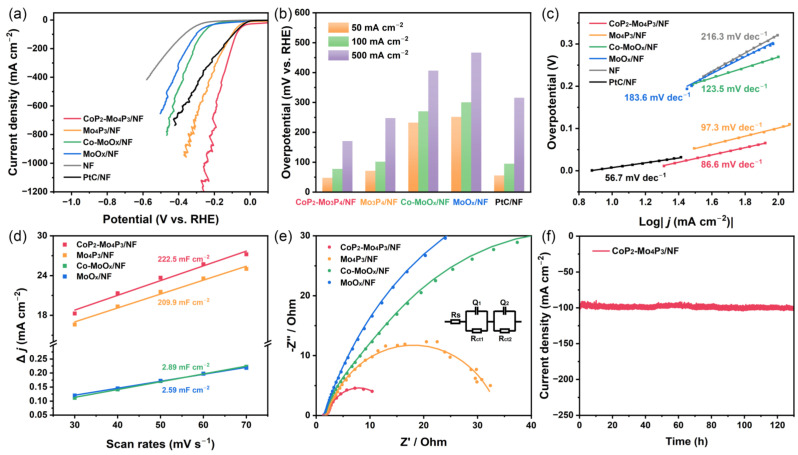
HER performance of synthesized catalysts in 1.0 M KOH: (**a**) LSV curves with iR-correction, (**b**) Overpotentials at different current densities of the obtained samples, (**c**) Corresponding Tafel plots derived from LSV, (**d**) Double-layer capacitance C_dl_, (**e**) EIS and its fitting patterns, (**f**) Stability measurements of CoP_2_-Mo_4_P_3_/NF catalyst at a current density of 100 mA cm^−2^.

The electrocatalytic performance of the catalysts for HER was evaluated by a typical three-electrode system in 1 M KOH electrolyte at room temperature. Figure 3a exhibits the LSV polarization curves (with 100% iR compensation) of the synthesized catalysts, and the catalytic activities of commercial 20 wt.% PtC/NF and NF were also tested for comparison. The CoP_2_-Mo_4_P_3_/NF catalyst reveals small overpotentials of 47.7, 77.6 and 170.7 mV at the current densities of 50, 100 and 500 mA cm^−2^, respectively, significantly better than those of MoO_x_/NF and Co-MoO_x_/NF precursors as well as Mo_4_P_3_/NF catalyst, and even superior to the commercial PtC/NF catalyst (Figure 3a,b). Particularly, at the overpotential of 265 mV, the CoP_2_-Mo_4_P_3_/NF catalyst exhibits a current density of 1200 mA cm^−2^, which is 3.4-fold higher than that of the commercial PtC/NF catalyst, demonstrating a significant enhancement of the HER catalytic activity. To gain insight into the HER kinetics of the prepared electrocatalysts, the Tafel slope was obtained by fitting the linear region of Tafel plots as shown in Figure 3c. The commercial PtC/NF exhibits an expected small Tafel slope of 56.7 mV dec^−1^, which is close to the value reported in the literature. CoP_2_-Mo_4_P_3_/NF catalyst reveals a smaller Tafel slope of 86.6 mV dec^−1^ compared to Mo_4_P_3_/NF (97.3 mV dec^−1^), Co-MoO_x_/NF (123.6 mV dec^−1^), MoO_x_/NF (183.6 mV dec^−1^) and NF (216.3 mV dec^−1^), indicating its faster kinetics for HER and the corresponding Volmer-Heyrovsky mechanism, where the reaction rate is limited by the Heyrovsky step (H_ads_ + H_3_O^+^ + e^−^ ⇌ H_2_ + H_2_O) [29]. Additionally, CoP_2_-Mo_4_P_3_/NF has a larger exchange current density (*j*_0_, calculated by the Tafel equation) of 14.71 mA cm^−2^ compared to that of PtC/NF (*j*_0_ = 7.38 mA cm^−2^), indicating its higher HER catalytic activity [14].

The electrochemical double-layer capacitance (C_dl_) values of the catalysts were also estimated from CV measurements in the non-Faradaic region at different scan rates of 30, 40, 50, 60 and 70 mV s^−1^, which are typically employed to calculate the electrochemically active surface areas (ECSA). The CV curves at different scan rates are shown in Figure S6 and the corresponding C_dl_ values are presented in Figure 3d. The CoP_2_-Mo_4_P_3_/NF catalyst exhibits the largest C_dl_ value of 222.5 mF cm^−2^ compared to MoO_x_/NF (2.59 mF cm^−2^), Co-MoO_x_/NF (2.89 mF cm^−2^) and Mo_4_P_3_/NF (209.9 mF cm^−2^), implying the largest ECSA of CoP_2_-Mo_4_P_3_/NF. This can be attributed to the small CoP_2_ nanoparticles on the surface of CoP_2_-Mo_4_P_3_/NF and the rich heterointerfaces between CoP_2_ and Mo_4_P_3_, leading to a high exposure of active sites. To further investigate the intrinsic activity, the turnover frequency (TOF) was also conducted according to our previous report [30]. Figure S7 reveals the calculated TOF of CoP_2_-Mo_4_P_3_/NF and Mo_4_P_3_/NF catalysts, and the TOF value of former reaches 1.19 s^−1^ at an over potential of 200 mV, much higher than that of Mo_4_P_3_/NF (0.74 s^−1^), indicating the superior intrinsic HER activity of CoP_2_-Mo_4_P_3_/NF catalyst.

The electrochemical impedance spectroscopy (EIS) measurements were carried out to evaluated the electrochemical resistances of the catalysts. The Nyquist plots of the EIS spectra, the corresponding fitted equivalent circuit model is presented in Figure 3e and the fitting parameters shown in Table S1. Obviously, the CoP_2_-Mo_4_P_3_/NF catalyst exhibits the smallest semicircle, indicating its lowest charge-transfer resistance (R_ct_) among the obtained catalysts (CoP_2_-Mo_4_P_3_/NF: 11.3 Ω, Mo_4_P_3_/NF: 32.2 Ω, Co-MoO_x_/NF: 81.6 Ω, MoO_x_/NF: 96.1 Ω). The low R_ct_ value of CoP_2_-Mo_4_P_3_/NF suggests the enhanced charge-transfer ability at the electrode/electrolyte interface, which is beneficial for the HER process. In addition, stability measurement was also performed by Chronoamperometry (CA) to assess the stability and durability of CoP_2_-Mo_4_P_3_/NF catalyst. As can be seen in Figure 3f, there was no significant decay after 130 h of HER test at a current density of 100 mA cm^−2^, demonstrating the outstanding stability of CoP_2_-Mo_4_P_3_/NF catalyst. Meanwhile, the SEM and TEM images of CoP_2_-Mo_4_P_3_/NF catalyst after HER stability experiment are displayed in Figure S8, which shows the similar morphology and composition with the initial, indicating its excellent structural stability during HER.

### 3.3. Electrocatalytic OER Performance

**Figure 4:**
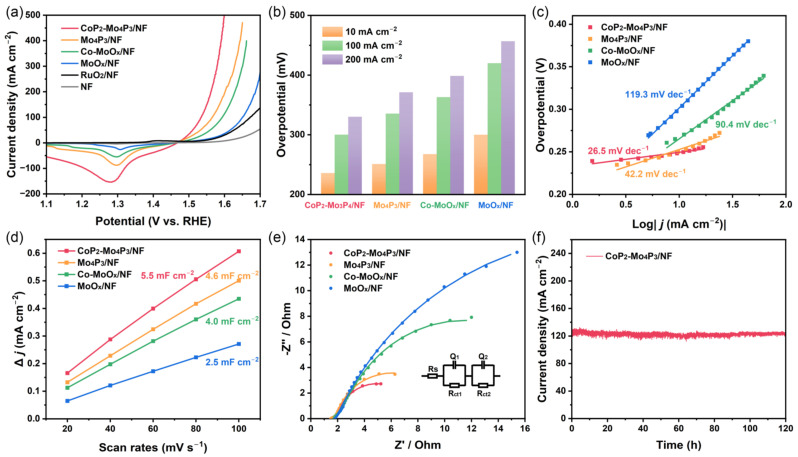
OER performance of synthesized catalysts in 1.0 M KOH: (**a**) LSV curves with iR-correction, (**b**) Overpotentials at different current densities of the obtained samples, (**c**) Corresponding Tafel plots derived from LSV, (**d**) Double-layer capacitance C_dl_, (**e**) EIS and its fitting patterns, (**f**) Stability measurements of CoP_2_-Mo_4_P_3_/NF catalyst at a current density of 125 mA cm^−2^.

The OER performance of the obtained catalysts was also evaluated using the three-electrode configuration in 1.0 M KOH electrolyte and the results are presented in Figure 4. CoP_2_-Mo_4_P_3_/NF catalyst shows the best OER performance among samples with the lowest overpotentials of 236, 300.3 and 330.2 mV at the current densities of 10, 100 and 200 mA cm^−2^, respectively, which is significantly lower than those of Mo_4_P_3_/NF, Co-MoO_x_/NF, MoO_x_/NF, RuO_2_/NF and NF (Figure 4b). In addition, the OER kinetics of the catalysts were analyzed by Tafel plot measurement (Figure 4c), where the Tafel slope value of CoP_2_-Mo_4_P_3_/NF catalyst was 26.5 mV dec^−1^, lower than those of Mo_4_P_3_/NF (42.2 mV dec^−1^), Co-MoO_x_/NF (90.4 mV dec^−1^), and MoO_x_/NF (119.3 mV dec^−1^), demonstrating its superior electrochemical kinetics for OER. The CV measurements at different scan rates of 20, 40, 60, 80 and 100 mV s^−1^ (Figure S9) were carried out to estimate the C_dl_ and ECSA of the synthesized catalysts. As shown in Figure 4d, CoP_2_-Mo_4_P_3_/NF exhibits a larger C_dl_ value of 5.5 mF cm^−2^ than those of Mo_4_P_3_/NF (4.6 mF cm^−2^), Co-MoO_x_/NF (4.0 mF cm^−2^) and MoO_x_/NF (2.5 mF cm^−2^). Accordingly, the ECSA of CoP_2_-Mo_4_P_3_/NF was calculated to be 137.5 cm^2^ (Figure S10), which is the highest among the prepared catalysts. This can be attributed to the structural peculiarity of CoP_2_-Mo_4_P_3_/NF, which consists of small sized CoP_2_ nanoparticles and rich heterointerfaces, thus creating more active sites. In addition, the specific activity normalized against ECSA was carried out to exclude the effect of surface area on the OER catalytic performance (Figure S11). It can be seen that the CoP_2_-Mo_4_P_3_/NF catalyst presents the best OER-specific activity among the obtained samples, indicating the excellent intrinsic OER activity of CoP_2_-Mo_4_P_3_/NF.

To further investigate the underlying reason for the enhanced OER activity of CoP_2_-Mo_4_P_3_/NF catalyst, the EIS measurements were also adopted and the Nyquist plots are presented in Figure 4e. Moreover, the fitted equivalent circuit is shown in the inset of Figure 4e and the corresponding fitted parameters are summarized in Table S2. Obviously, the CoP_2_-Mo_4_P_3_/NF catalyst also exhibits a lower R_ct_ value of 7.57 Ω than Mo_4_P_3_/NF (9.59 Ω), Co-MoO_x_/NF (21.2 Ω) and MoO_x_/NF (44.2 Ω), indicating its faster charge transfer kinetics at the electrode/electrolyte interface, which can improve the intrinsic activity of OER. Figure 4f shows the result of electrochemical stability test performed by CA measurement. The OER catalytic performance of CoP_2_-Mo_4_P_3_/NF catalyst reveals neglectable degradation at the end of 120 h OER test at a high current density of 125 mA cm^−2^. Additionally, the SEM and TEM images (Figure S12) of CoP_2_-Mo_4_P_3_/NF catalyst after OER stability measurement still demonstrate the same morphology and structure as the initial, suggesting its superior stability for OER.

### 3.4. Overall Water Splitting

**Figure 5:**
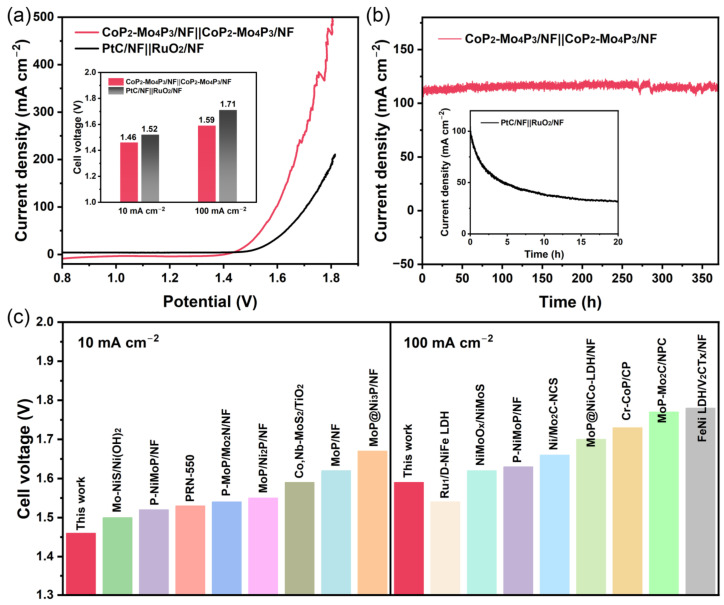
(**a**) LSV polarization curves for the CoP_2_-Mo_4_P_3_/NF||CoP_2_-Mo_4_P_3_/NF and PtC/NF||RuO_2_/NF in a 1.0 M KOH electrolyte. Inset: comparison of the driving voltages required to reach different current densities for different catalysts, (**b**) Stability measurements of CoP_2_-Mo_4_P_3_/NF||CoP_2_-Mo_4_P_3_/NF and PtC/NF||RuO_2_/NF (inset), (**c**) Cell voltage comparison of CoP_2_-Mo_4_P_3_/NF||CoP_2_-Mo_4_P_3_/NF with recently reported state-of-the-art OWS catalysts.

Based on the above results, the obtained CoP_2_-Mo_4_P_3_/NF catalyst exhibits excellent catalytic activity and stability for both HER and OER, which is practically usable for the overall water splitting (OWS). Therefore, a two-electrode electrolyzer using CoP_2_-Mo_4_P_3_/NF catalyst as both cathodic HER and anodic OER catalysts (marked as CoP_2_-Mo_4_P_3_/NF||CoP_2_-Mo_4_P_3_/NF) was assembled to evaluate the practical usability of CoP_2_-Mo_4_P_3_/NF catalyst toward the OWS in 1.0 M KOH electrolyte. For comparison, the commercial PtC/NF and RuO_2_/NF were, respectively, used as cathodic and anodic catalysts (marked as PtC/NF||RuO_2_/NF) to assemble the two-electrode electrolyzer and test the performance of OWS. The polarization curves in Figure 5a show that CoP_2_-Mo_4_P_3_/NF||CoP_2_-Mo_4_P_3_/NF exhibits higher performance for OWS with low cell voltages of 1.46 and 1.59 V to achieve current densities of 10 and 100 mA cm^−2^, respectively, which is superior to PtC/NF||RuO_2_/NF (1.52 and 1.71 V to achieve current densities of 10 and 100 mA cm^−2^, respectively). Moreover, it requires a much lower cell voltage of 1.80 V for CoP_2_-Mo_4_P_3_/NF||CoP_2_-Mo_4_P_3_/NF to achieve a high current density of 500 mA cm^−2^. Additionally, the CoP_2_-Mo_4_P_3_/NF||CoP_2_-Mo_4_P_3_/NF displays no obvious degradation in its OWS performance after 370 h at a high current density of 110 mA cm^−2^, while PtC/NF||RuO_2_/NF retains only 30 % of its initial current density (100 mA cm^−2^) after 20 h (Figure 5b). To the best of our knowledge, the CoP_2_-Mo_4_P_3_/NF||CoP_2_-Mo_4_P_3_/NF electrolyzer shows comparable and even superior electrocatalytic activity to the recently reported state-of-the-art catalysts as presented in Figure 5c and Table S3. The above results indicate that the CoP_2_-Mo_4_P_3_/NF catalyst can meet the requirements of water splitting with high current density and being stable in industrial applications.

## 4. Conclusions

In summary, CoP_2_-Mo_4_P_3_/NF catalyst with rich heterointerfaces was successfully prepared by a two-step hydrothermal-phosphorylation method, which was proved to be an efficient bifunctional catalyst for the overall water splitting in alkaline electrolyte. The synergistic interaction between CoP_2_ and Mo_4_P_3_ heterogeneous interfaces can optimize the electronic structure, weaken the adsorption of H on the Mo sites and increase the redox activity of the Co site, leading to the enhancement of bifunctional catalytic activities of the CoP_2_-Mo_4_P_3_/NF catalyst toward HER and OER. Accordingly, the CoP_2_-Mo_4_P_3_/NF catalyst exhibits desirable bifunctional electrocatalytic performance for HER and OER under alkaline conditions, with low overpotentials of 77.6 and 300.3 at 100 mA cm^−2^ for HER and OER, respectively. Additionally, the CoP_2_-Mo_4_P_3_/NF||CoP_2_-Mo_4_P_3_/NF electrolyzer also displays excellent catalytic activity and stability for the overall water splitting in a two-electrode system, which can achieve 100 mA cm^−2^ at the cell voltages of 1.59 V and keep stable for at least 370 h at a high current density of 110 mA cm^−2^, indicating that the CoP_2_-Mo_4_P_3_/NF catalyst can meet the requirements of water splitting with high current density and stability in industrial applications. This work provides a novel inspiration for the design of highly efficient and cost effective bifunctional electrocatalysts for the overall water splitting.

## Data Availability

Not applicable.

## Supplementary Materials

The following supporting information can be downloaded at: https://www.mdpi.com/article/10.3390/nano13010074/s1. Figure S1: The XRD patterns of (a) Co-MoO_x_/NF and MoO_x_/NF precursors, and (b) CoP_2_-Mo_4_P_3_/NF and Mo_4_P_3_/NF, Figure S2: SEM images of (a) MoO_x_/NF and (b) Co-MoO_x_/NF precursors, Figure S3: SEM images of Mo_4_P_3_/NF catalyst at different magnifications, Figure S4: TEM-EDS line scanning image of CoP_2_-Mo_4_P_3_/NF (left) and the corresponding elemental spectra (right), Figure S5: (a, b) TEM, (c) HRTEM, (d) SAED and (e) the corresponding elemental mapping images of Mo_4_P_3_/NF catalyst, Figure S6: CV curves of (a) CoP_2_-Mo_4_P_3_/NF, (b) Mo_4_P_3_/NF, (c) Co-MoO_x_/NF and (d) MoO_x_/NF at potential regions of 0.29-0.39 V (vs. RHE) with varied scan rates of 30-70 mV s^−1^ in 1.0 M KOH, Figure S7: TOF curves of CoP_2_-Mo_4_P_3_/NF and Mo_4_P_3_/NF catalysts, Figure S8: (a, b) SEM and (c, d) TEM images of CoP_2_-Mo_4_P_3_/NF catalyst after HER stability experiment, Figure S9: CV curves of (a) CoP_2_-Mo_4_P_3_/NF, (b) Mo_4_P_3_/NF, (c) Co-MoO_x_/NF and (d) MoO_x_/NF at potential regions of 1.028-1.128 V (vs. RHE) with varied scan rates of 20-100 mV s^−1^ in 1.0 M KOH, Figure S10: The calculated ECSA values of synthesized catalysts, Figure S11: LSV curves normalized against ECSA, Figure S12: (a, b) SEM and (c, d) TEM images of CoP_2_-Mo_4_P_3_/NF catalyst after OER stability experiment, Table S1: EIS parameters of synthesized catalysts in 1.0 M KOH for HER, Table S2: EIS parameters of synthesized catalysts in 1.0 M KOH for OER, Table S3: Comparison of CoP_2_-Mo_4_P_3_/NF||CoP_2_-Mo_4_P_3_/NF with recently reported state-of-the-art OWS catalysts. References [11,12,13,14,15,17,18,25,31,32,33,34,35,36,37] are cited in the Supplementary Materials.
