# Supplementary material for: Construction of CoP2-Mo4P3/NF Heterogeneous Interfacial Electrocatalyst for Boosting Water Splitting
Source: Nanomaterials (Basel). 2022 Dec 23;13(1):74. doi: 10.3390/nano13010074 (PMC9824638; doi:10.3390/nano13010074)
Supplement: Supplementary file 1 [file nanomaterials-13-00074-s001.zip › nanomaterials-2107522-supplementary.pdf]

Construction of CoP_2-Mo_4P_3/NF Heterogeneous Interfacial Electrocatalyst for Boosting Water Splitting

Yafeng Chen [1,2], Ge Meng [1], Ziwei Chang [1], Ningning Dai [3], Chang Chen [1], Xinmei Hou [2,*] and Xiangzhi Cui [1,4,*]

1 The State Key Laboratory of High Performance Ceramics and Superfine Microstructures, Shanghai Institute of Ceramics, Chinese Academy of Sciences, Shanghai 200050, China
2 Beijing Advanced Innovation Center for Materials Genome Engineering, Collaborative Innovation Center of Steel Technology, University of Science and Technology Beijing, Beijing 100083, China
3 Shanghai Motor Vehicle Inspection Certification & Tech Innovation Center Co., Ltd., Shanghai 201805, China
4 School of Chemistry and Materials Science, Hangzhou Institute for Advanced Study, University of Chinese Academy of Sciences, Hangzhou 310021, China
* Correspondence: houxinmeiustb@ustb.edu.cn (X.H.)；cuixz@mail.sic.ac.cn (X.C.)

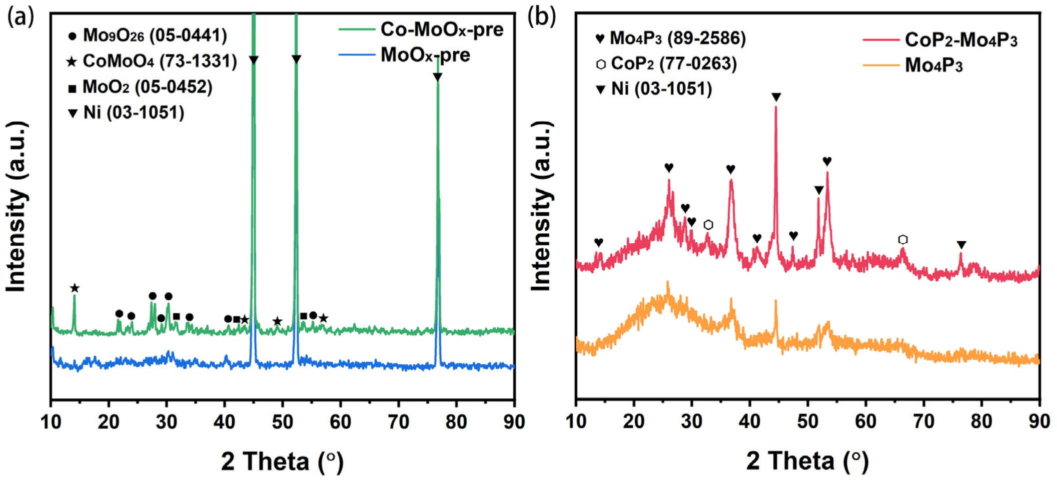

Figure S1. The XRD patterns of (a) Co-MoO_x/NF and MoO_x/NF precursors, and (b) CoP_2-Mo_4P_3/NF and Mo_4P_3/NF.

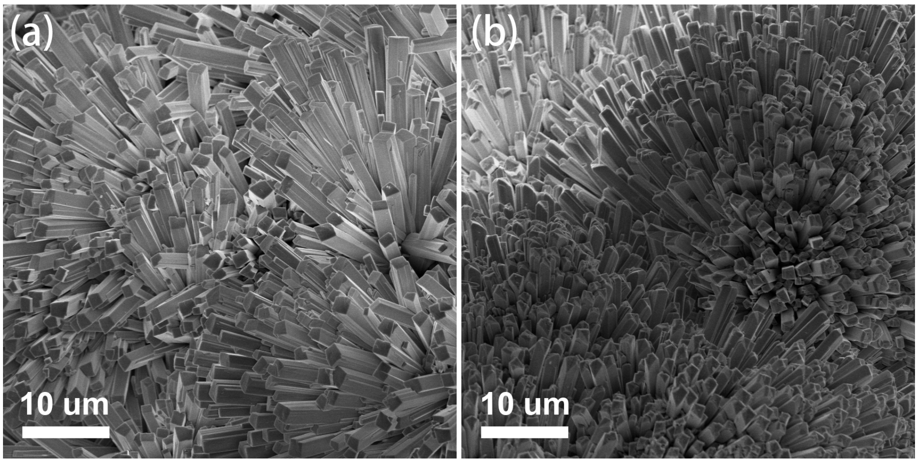

Figure S2. SEM images of (a) MoO_x/NF and (b) Co-MoO_x/NF precursors.

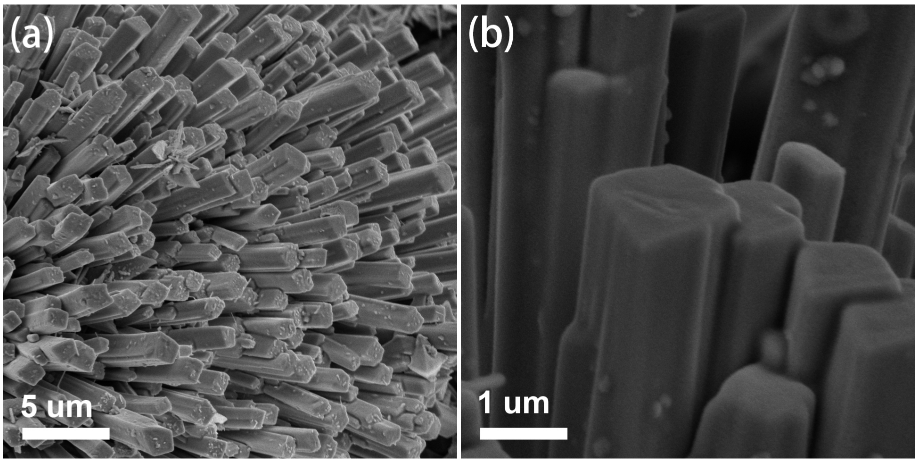

Figure S3. SEM images of Mo_4P_3/NF catalyst at different magnifications.

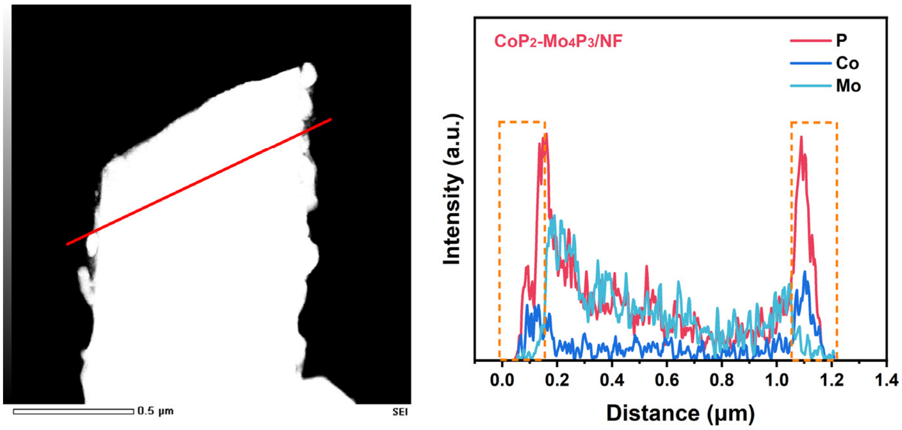

Figure S4. TEM-EDS line scanning image of CoP_2-Mo_4P_3/NF (left) and the corresponding elemental spectra (right).

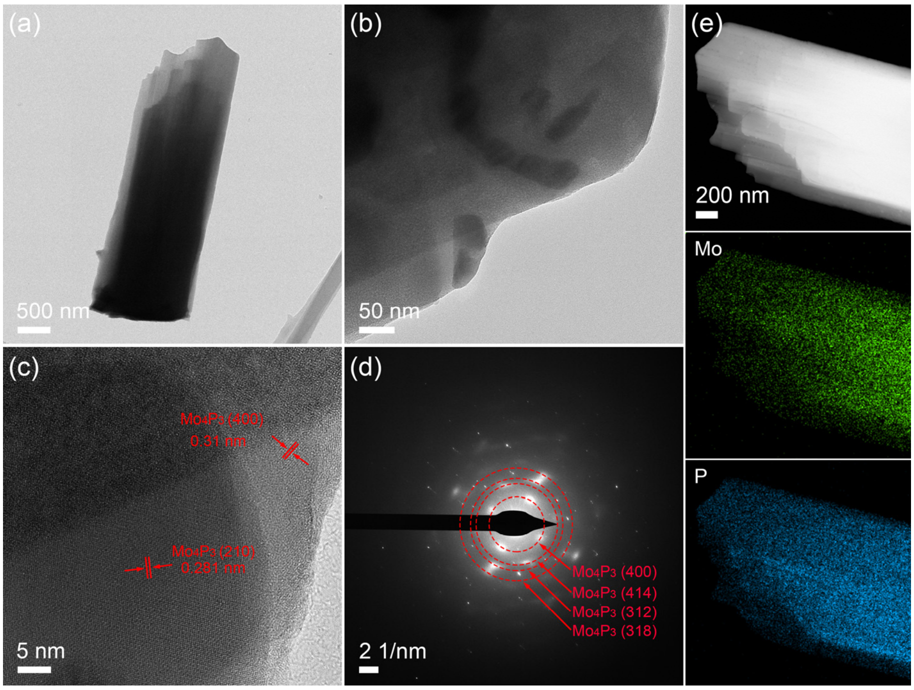

Figure S5. (a, b) TEM, (c) HRTEM, (d) SAED and (e) the corresponding elemental mapping images of Mo_4P_3/NF catalyst.

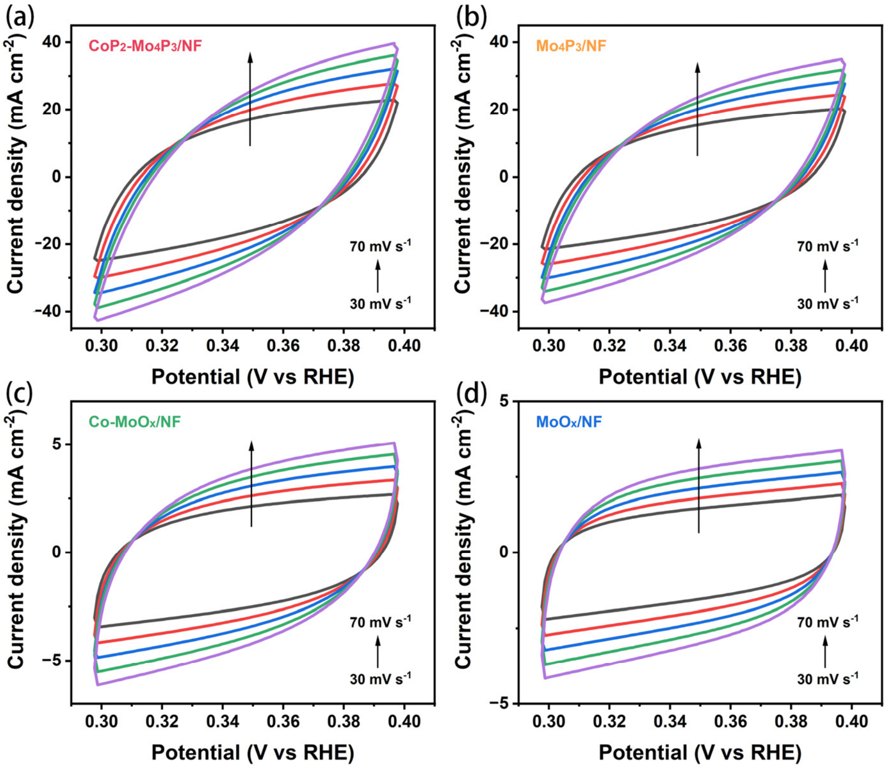

Figure S6. CV curves of (a) CoP_2-Mo_4P_3/NF, (b) Mo_4P_3/NF, (c) Co-MoO_x/NF and (d) MoO_x/NF at potential regions of 0.29-0.39 V (vs. RHE) with varied scan rates of 30-70 mV s^{-1} in 1.0 M KOH.

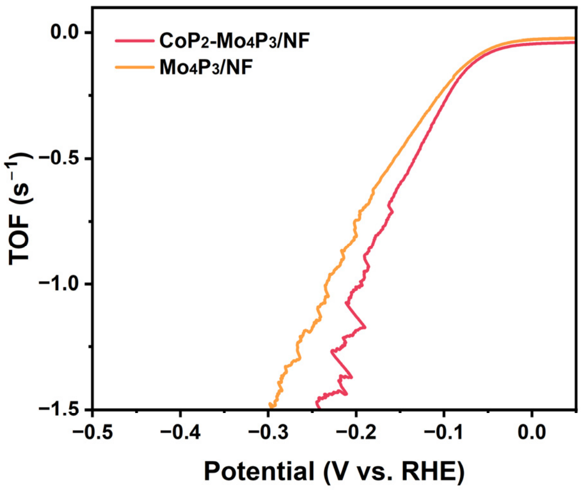

Figure S7. TOF curves of CoP_2-Mo_4P_3/NF and Mo_4P_3/NF catalysts.

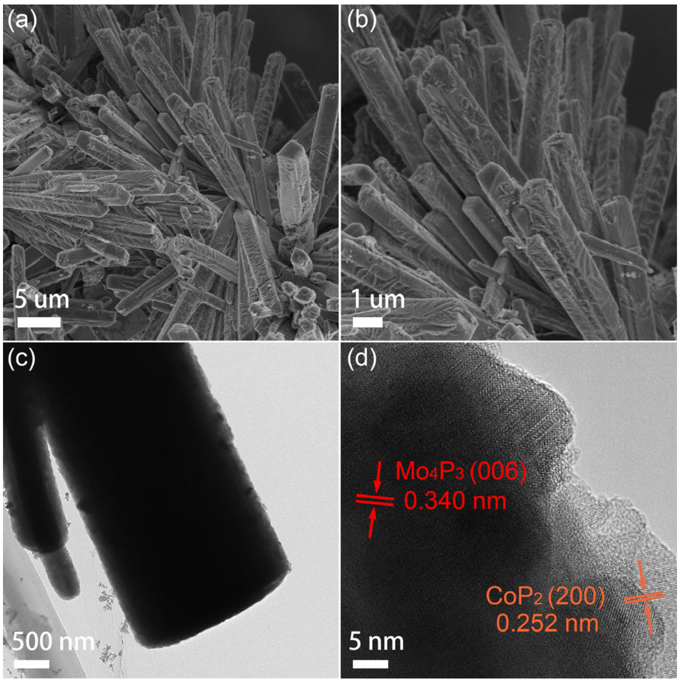

Figure S8. (a, b) SEM and (c, d) TEM images of CoP_2-Mo_4P_3/NF catalyst after HER stability experiment.

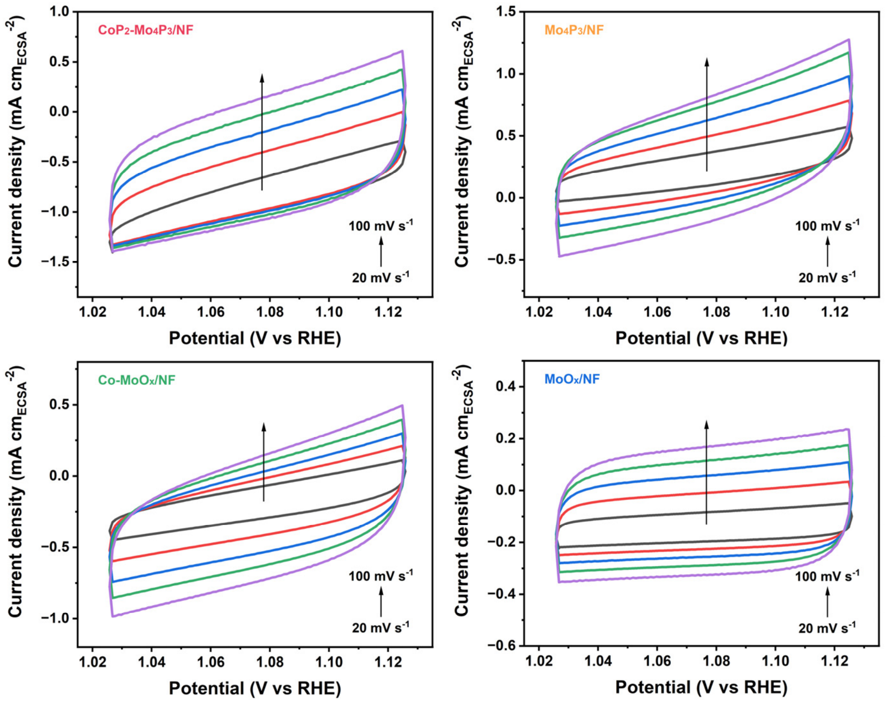

Figure S9. CV curves of (a) CoP_2-Mo_4P_3/NF, (b) Mo_4P_3/NF, (c) Co-MoO_x/NF and (d) MoO_x/NF at potential regions of 1.028-1.128 V (vs. RHE) with varied scan rates of 20-100 mV s^{-1} in 1.0 M KOH.

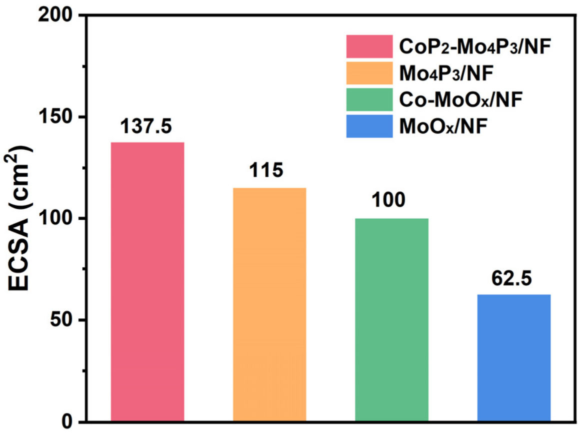

Figure S10. The calculated ECSA values of synthesized catalysts.

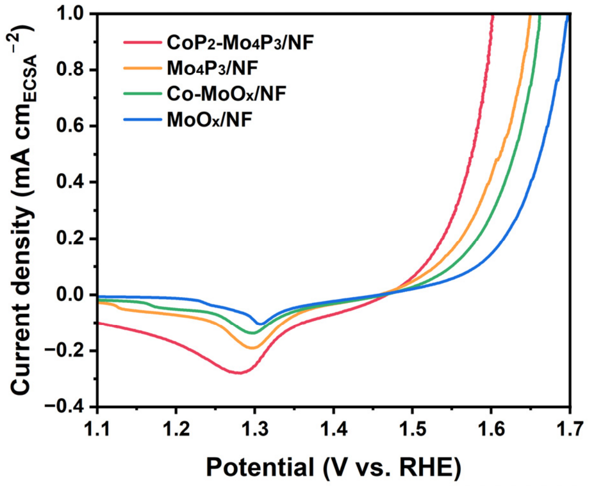

Figure S11. LSV curves normalized against ECSA.

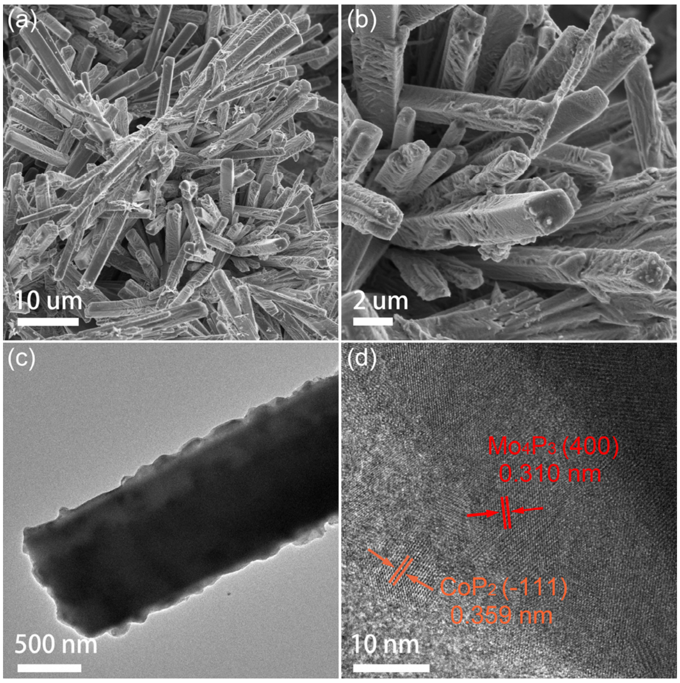

Figure S12. (a, b) SEM and (c, d) TEM images of CoP_2-Mo_4P_3/NF catalyst after OER stability experiment.

Table S1. EIS parameters of synthesized catalysts in 1.0 M KOH for HER.

Materials	Rs(Ω)	Q_1(S·s^n)	n_1	R_{ct1}(Ω)	Q_2(S·s^n)	n_2	R_{ct2}(Ω)
**CoP_2-Mo_4P_3/NF**	1.664	0.004	0.8	4.15	0.002	0.8	11.3
**Mo_4P_3/NF**	1.631	0.002	0.8	0.54	0.004	0.8	32.2
**Co-MoO_x/NF**	1.674	0.001	0.9	5.19	0.001	0.8	81.6
**MoO_x/NF**	1.584	0.005	0.8	2.69	0.001	0.8	96.1

Table S2. EIS parameters of synthesized catalysts in 1.0 M KOH for OER.

**Materials**	**Rs(Ω)**	**$Q_1(S \cdot s^n)$**	**n_1**	**$R_{ct1}(Ω)$**	**$Q_2(S \cdot s^n)$**	**n_2**	**$R_{ct2}(Ω)$**
**CoP_2-Mo_4P_3/NF**	1.514	0.096	0.9	0.49	0.295	0.67	7.57
**Mo_4P_3/NF**	1.492	0.197	0.7	0.42	0.839	0.89	9.59
**Co-MoO_x/NF**	1.674	0.200	0.8	0.12	0.260	0.80	21.2
**MoO_x/NF**	1.741	0.128	0.8	0.44	0.161	0.74	44.2

Table S3. Comparison of CoP_2-Mo_4P_3/NF||CoP_2-Mo_4P_3/NF with recently reported state-of-the-art OWS catalysts.

Catalysts	Electrolyte	Cell voltage (V@j(mA cm^{-2}))	Ref
**CoP_2-Mo_4P_3/NF**	1.0 M KOH	1.46@10<br>1.59@100	This work
**Mo-NiS/$Ni(OH)_2$**	1.0 M KOH	1.50@10	[1]
**P-NiMoP/NF**	1.0 M KOH	1.52@10	[2]
**PRN-550**	1.0 M KOH	1.53@10	[3]
**P-MoP/Mo_2N/NF**	1.0 M KOH	1.54@10	[4]
**MoP/Ni_2P/NF**	1.0 M KOH	1.55@10	[5]
**Co,Nb-MoS_2/TiO_2 HSs**	1.0 M KOH	1.59@10	[6]
**MoP/NF**	1.0 M KOH	1.62@10	[7]
**MoP@Ni_3P/NF**	1.0 M KOH	1.67@10	[8]
**Ru_1/D-NiFe LDH**	1.0 M KOH	1.54@100	[9]
**NiMoOx/NiMoS**	1.0 M KOH	1.62@100	[10]
**P-NiMoP/NF**	1.0 M KOH	1.63@100	[2]
**Ni/Mo_2C-NCS**	1.0 M KOH	1.66@100	[11]
**MoP@NiCo-LDH/NF**	1.0 M KOH	1.7@100	[12]
**Cr-CoP/CP**	1.0 M KOH	1.73@100	[13]
**MoP-Mo_2C/NPC**	1.0 M KOH	1.77@100	[14]
**FeNi LDH/V_2CTx/NF**	1.0 M KOH	1.78@100	[15]

References

1. Zhang, H.; Xi, B.; Gu, Y.; Chen, W.; Xiong, S. Interface engineering and heterometal doping Mo-NiS/Ni(OH)$_2$ for overall water splitting. *Nano Res.* **2021**, *14*, 3466-3473.
2. Zhang, B.; Jiang, Z.; Shang, X.; Li, S.; Jiang, Z.-J. Accelerated hydrogen evolution reaction in Ni$_3$P/MoP$_2$/MoO$_2$ tri-phase composites with rich crystalline interfaces and oxygen vacancies achieved by plasma assisted phosphorization. *J. Mater. Chem. A* **2021**, *9*, 25934-25943.
3. Kim, M.; Park, J.; Ju, H.; Kim, J. Y.; Cho, H.-S.; Kim, C.-H.; Kim, B.-H.; Lee, S. W. Understanding synergistic metal–oxide interactions of in situ exsolved metal nanoparticles on a pyrochlore oxide support for enhanced water splitting. *Energy Environ. Sci.* **2021**, *14*, 3053-3063.
4. Gu, Y.; Wu, A.; Jiao, Y.; Zheng, H.; Wang, X.; Xie, Y.; Wang, L.; Tian, C.; Fu, H. Two-Dimensional Porous Molybdenum Phosphide/Nitride Heterojunction Nanosheets for pH-Universal Hydrogen Evolution Reaction. *Angew Chem Int Ed Engl* **2021**, *60*, 6673-6681.
5. Du, C.; Shang, M.; Mao, J.; Song, W. Hierarchical MoP/Ni$_2$P heterostructures on nickel foam for efficient water splitting. *J. Mater. Chem. A* **2017**, *5*, 15940-15949.
6. Nguyen, D. C.; Luyen Doan, T. L.; Prabhakaran, S.; Tran, D. T.; Kim, D. H.; Lee, J. H.; Kim, N. H. Hierarchical Co and Nb dual-doped MoS$_2$ nanosheets shelled micro-TiO$_2$ hollow spheres as effective multifunctional electrocatalysts for HER, OER, and ORR. *Nano Energy* **2021**, *82*, 105750.
7. Jiang, Y.; Lu, Y.; Lin, J.; Wang, X.; Shen, Z. A Hierarchical MoP Nanoflake Array Supported on Ni Foam: A Bifunctional Electrocatalyst for Overall Water Splitting. *Small Methods* **2018**, *2*, 1700369.
8. Wang, F.; Chen, J.; Qi, X.; Yang, H.; Jiang, H.; Deng, Y.; Liang, T. Increased nucleation sites in nickel foam for the synthesis of MoP@Ni$_3$P/NF nanosheets for bifunctional water splitting. *Appl. Surf. Sci.* **2019**, *481*, 1403-1411.
9. Zhai, P.; Xia, M.; Wu, Y.; Zhang, G.; Gao, J.; Zhang, B.; Cao, S.; Zhang, Y.; Li, Z.; Fan, Z.; Wang, C.; Zhang, X.; Miller, J. T.; Sun, L.; Hou, J. Engineering single-atomic ruthenium catalytic sites on defective nickel-iron layered double hydroxide for overall water splitting. *Nat. Commun.* **2021**, *12*, 4587.
10. Zhai, P.; Zhang, Y.; Wu, Y.; Gao, J.; Zhang, B.; Cao, S.; Zhang, Y.; Li, Z.; Sun, L.; Hou, J. Engineering active sites on hierarchical transition bimetal oxides/sulfides heterostructure array enabling robust overall water splitting. *Nat. Commun.* **2020**, *11*, 5462.
11. Xu, Y.; Yang, J.; Liao, T.; Ge, R.; Liu, Y.; Zhang, J.; Li, Y.; Zhu, M.; Li, S.; Li, W. Bifunctional water splitting enhancement by manipulating Mo-H bonding energy of transition Metal-Mo$_2$C heterostructure catalysts. *Chem. Eng. J.* **2022**, *431*, 134126.

12. Wang, T.; Wu, H.; Feng, C.; Zhang, L.; Zhang, J. MoP@NiCo-LDH on nickel foam as bifunctional electrocatalyst for high efficiency water and urea–water electrolysis. *J. Mater. Chem. A* **2020**, *8*, 18106-18116.
13. Li, W.; Jiang, Y.; Li, Y.; Gao, Q.; Shen, W.; Jiang, Y.; He, R.; Li, M. Electronic modulation of CoP nanoarrays by Cr-doping for efficient overall water splitting. *Chem. Eng. J.* **2021**, *425*, 130651.
14. Jiang, E.; Li, J.; Li, X.; Ali, A.; Wang, G.; Ma, S.; Kang Shen, P.; Zhu, J. MoP-Mo_2C quantum dot heterostructures uniformly hosted on a heteroatom-doped 3D porous carbon sheet network as an efficient bifunctional electrocatalyst for overall water splitting. *Chem. Eng. J.* **2022**, *431*, 133719.
15. Yang, L.; Yang, T.; Chen, Y.; Zheng, Y.; Wang, E.; Du, Z.; Chou, K. C.; Hou, X. FeNi LDH/V_2CT_x/NF as Self-Supported Bifunctional Electrocatalyst for Highly Effective Overall Water Splitting. *Nanomaterials (Basel)* **2022**, *12*. 2640.
